# Supplementary material for: Surface restructuring of a perovskite-type air electrode for reversible protonic ceramic electrochemical cells
Source: Nat Commun. 2022 Apr 22;13:2207. doi: 10.1038/s41467-022-29866-5 (PMC9033792; doi:10.1038/s41467-022-29866-5)
Supplement: Supplementary file 1 — Supplementary Information [file 41467_2022_29866_MOESM1_ESM.pdf]

SUPPLEMENTARY INFORMATION

Surface Restructuring of a Perovskite-type Air Electrode for Reversible Protonic Ceramic Electrochemical Cells

Kai Pei[1#], Yucun Zhou[2#], Kang Xu[#1], Hua Zhang[1], Yong Ding[2], Bote Zhao[1], Wei Yuan[2,3], Kotaro Sasaki[4], YongMan Choi[5]*, Yu Chen[1,6]*, and Meilin Liu[2]*

[1]School of Environment and Energy, South China University of Technology, Guangzhou, China

[2]School of Materials Science and Engineering, Georgia Institute of Technology, Atlanta, GA 30309, USA

[3]School of Mechanical and Automotive Engineering, South China University of Technology, Guangzhou, 510640, China

[4]Chemistry Department, Brookhaven National Laboratory, Upton, NY 11973, USA

[5]College of Photonics, National Yang Ming Chiao Tung University, Tainan 71150, Taiwan

[6]Guangdong Provincial Key Laboratory of Atmospheric Environment and Pollution Control, South China University of Technology, Guangzhou Higher Education Mega Centre, Guangzhou 510006, PR China

[#] These authors contribute equally: Kai Pei, Yucun Zhou, and Kang Xu

*Corresponding authors:

YongMan Choi (ymchoi@nctu.edu.tw);

Yu Chen (eschenyu@scut.edu.cn);

Meilin Liu (Meilin.liu@mse.gatech.edu).

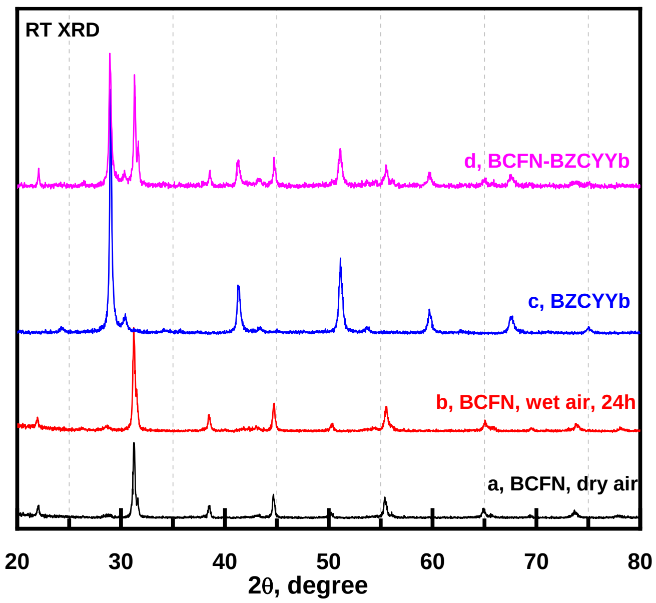

Supplementary Figure 1. XRD patterns of as-prepared BCFN powder **(a)**, BCFN powder treated in wet air (3 %) for 24 h at 700ºC **(b)**, BZCYYb **(c),** and mixed BZCYYb-BCFN (1:1 wt.%) **(d)** powders after calcining at 1000 ºC for 2 h in air.

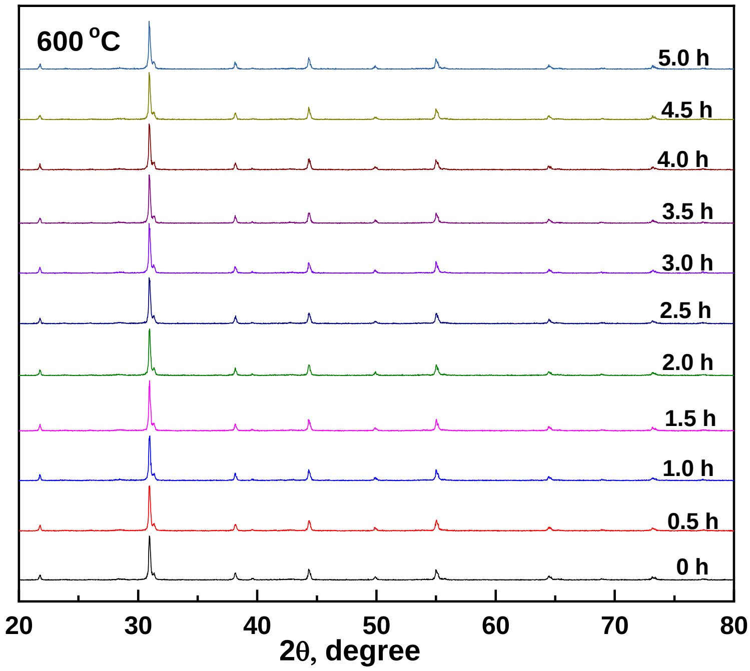

Supplementary Figure 2. In situ XRD patterns of BCFN powder in wet air (3% H_2O) at 600°C for 5 h. The data were collected every 30 min.

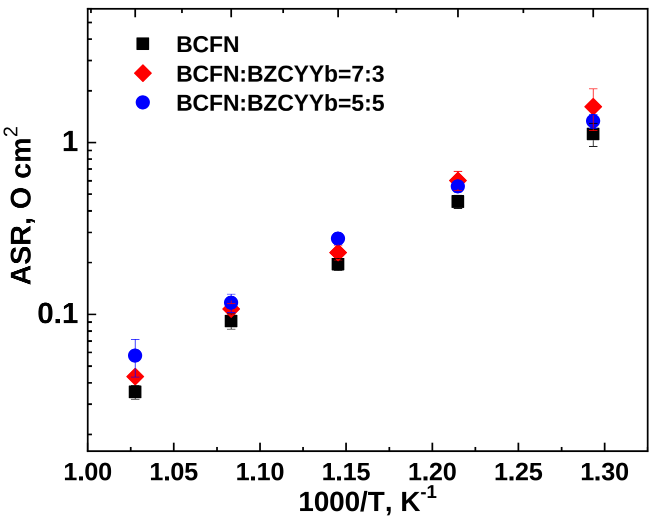

Supplementary Figure 3 Temperature dependence of the polarization resistance (Rp) of symmetrical cell with BCFN and composite electrodes

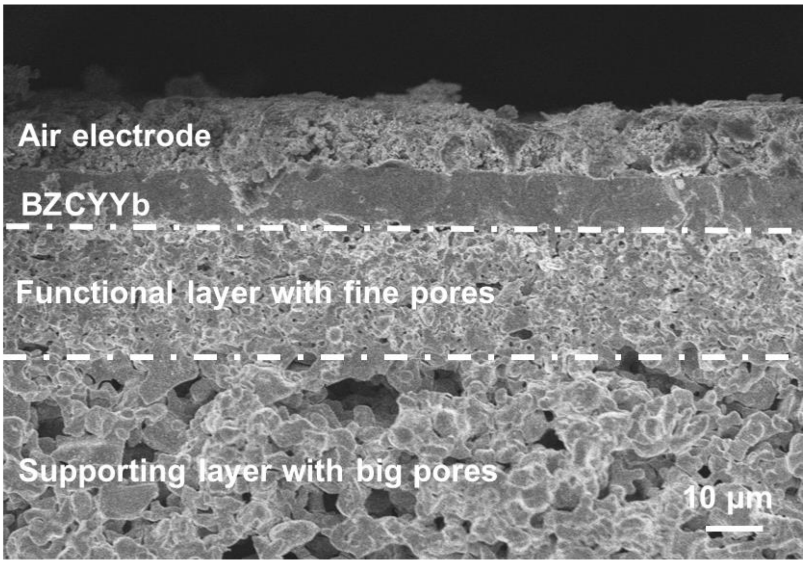

Supplementary Figure 4. Typical SEM image of a single cell with distinct four layers.

Supplementary Discussion: As shown, a multi-layered structure, including a porous Ni-BZCYYb anode supporting layer (ASL, ~600 μm), a porous but fine Ni-BZCYYb anode functional layer (AFL, ~25 μm), a dense BZCYYb electrolyte layer (~10 μm), and a porous BCFN layer (~15 μm), were adhered well with no cracks or delamination **(Supplementary Figure 4)**. The functional layer has finer pores and a larger surface area (due mainly to the reduction of NiO), providing more triple-phase boundaries for electrochemical reactions. In contrast, the supporting layer has larger pores and continuous channels (due mainly to the removal of pore-former), providing facile paths for gas transport.

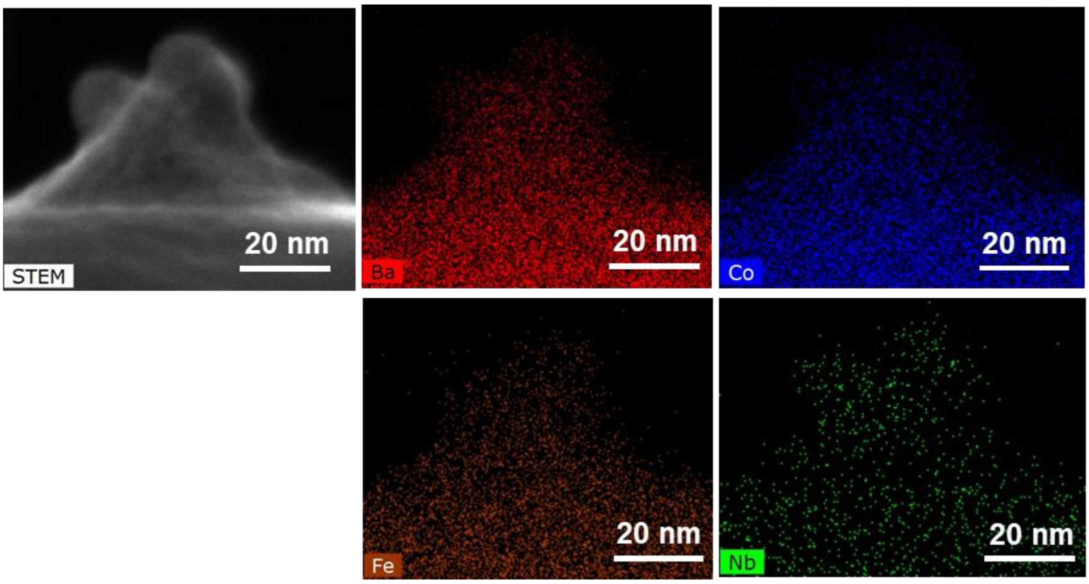

Supplementary Figure 5. STEM image of BCFN powder before the electrochemical test and the mapping of Ba, Co, Fe, Nb. The image shows a uniform element distribution.

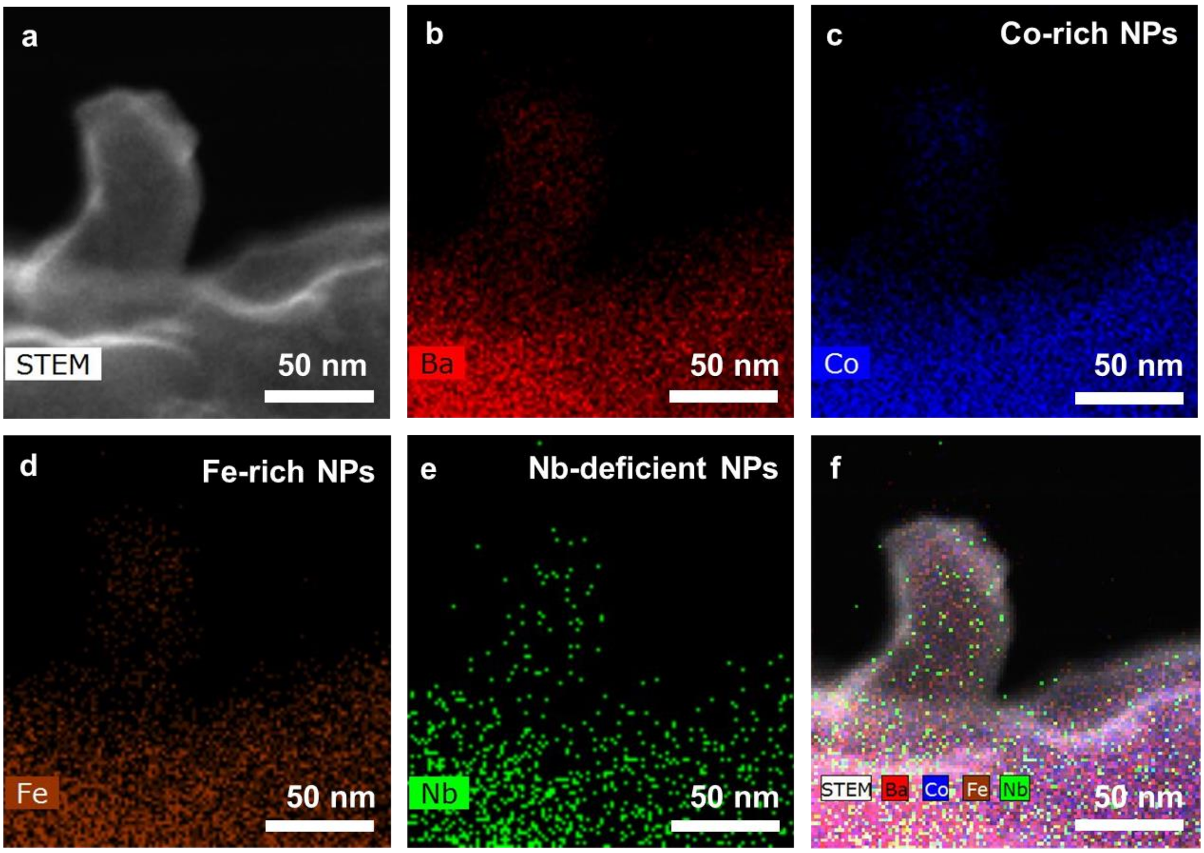

Supplementary Figure 6 An elemental mapping of BCFN grain covered with a nanoparticle (a), and the mapping of Ba (b), Co (c), Fe (d), Nb (e), and all (f).

Supplementary Discussion: We performed elemental mappings of Ba, Co, Fe, and Nb of a BCFN grain (after test) covered with a nanoparticle, as shown in **Supplementary Figure 6**. It is confirmed that there is likely a Co/Fe segregation within the nanoparticle.

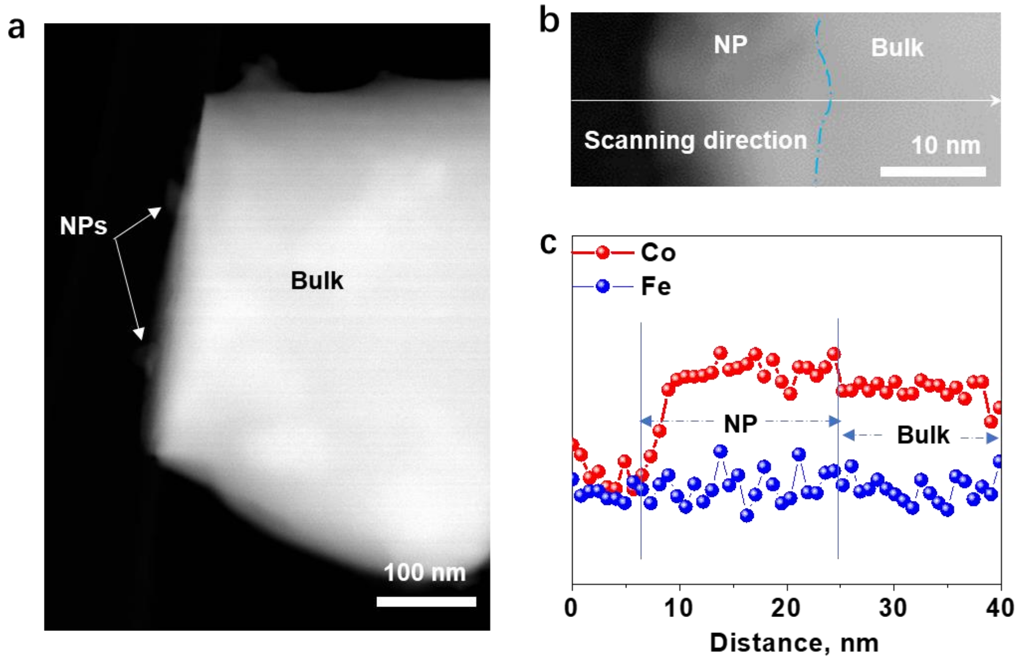

Supplementary Figure 7 (a) and **(b)** A STEM image of a BCFN grain covered with nanoparticles; and **(c)** an electron energy-loss spectroscopy (EELS) profile along the white line shown in (a).

Supplementary Discussion: Shown in **Supplementary Figures 7a** and **7b** are the STEM images of a BCFN grain covered with nanoparticles. Shown in **Supplementary Figure 7c** is the electron energy-loss spectroscopy (EELS) profile of Co and Fe along the scanning line shown in **Supplementary Figure 7b**. Slightly higher contents of Co or Fe are observed in the EELS profile (**Supplementary Figure 7c**), suggesting a possible Co/Fe segregation.

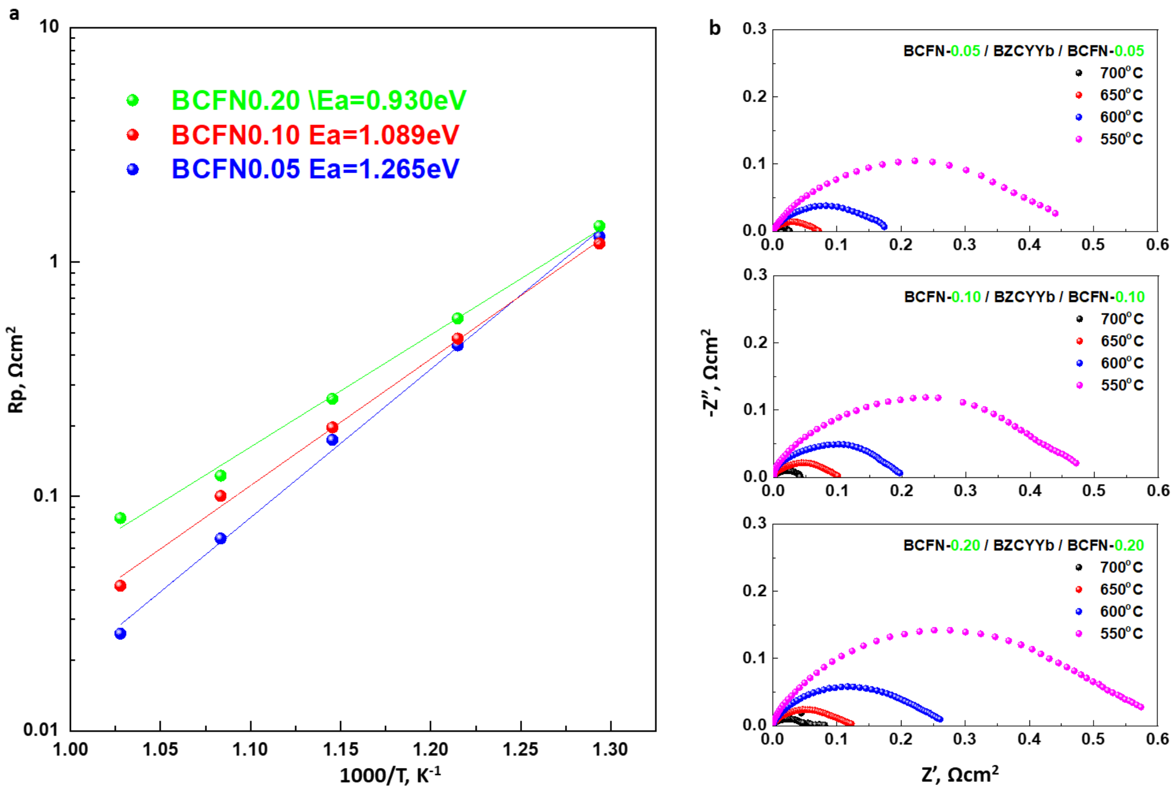

Supplementary Figure 8 (a) Arrhenius plots of the area-specific resistance (ASR, noted as Rp) for BCFN with different Nb content, measured from 700 to 500 °C, in flowing air (20 sccm) with 3% H_2O. **(b)** Detailed Temperature dependence of the polarization resistance (R_p) of symmetrical cell with BCFN ($Ba_{0.9}Co_{0.7}Fe_{0.2}Nb_xO_{3-\delta}$) with Nb content of 0.05, 0.10, 0.20, respectively. The electrode with 5% Nb doping at B-site showed the lowest R_p at 700-550°C, indicating that BCFN with less Nb shows a higher ORR activity.

Supplementary Discussion: A relatively higher Ea value for Nb deficient BCFN was observed, suggesting that there is a higher reaction barrier has to be overcome in the oxygen reaction process. It is shown that Rp values of BCFN0.05, BCFN0.1, and BCFN0.2 were similar at a low temperature of 550 °C. The difference became more pronounced as the operating temperature increased: Nb-deficient BCFN showed a lower Rp value, which is likely caused by the more oxygen vacancy formed at higher temperatures. Nb deficient BCFN is expected to have more oxygen vacancy at higher temperatures.

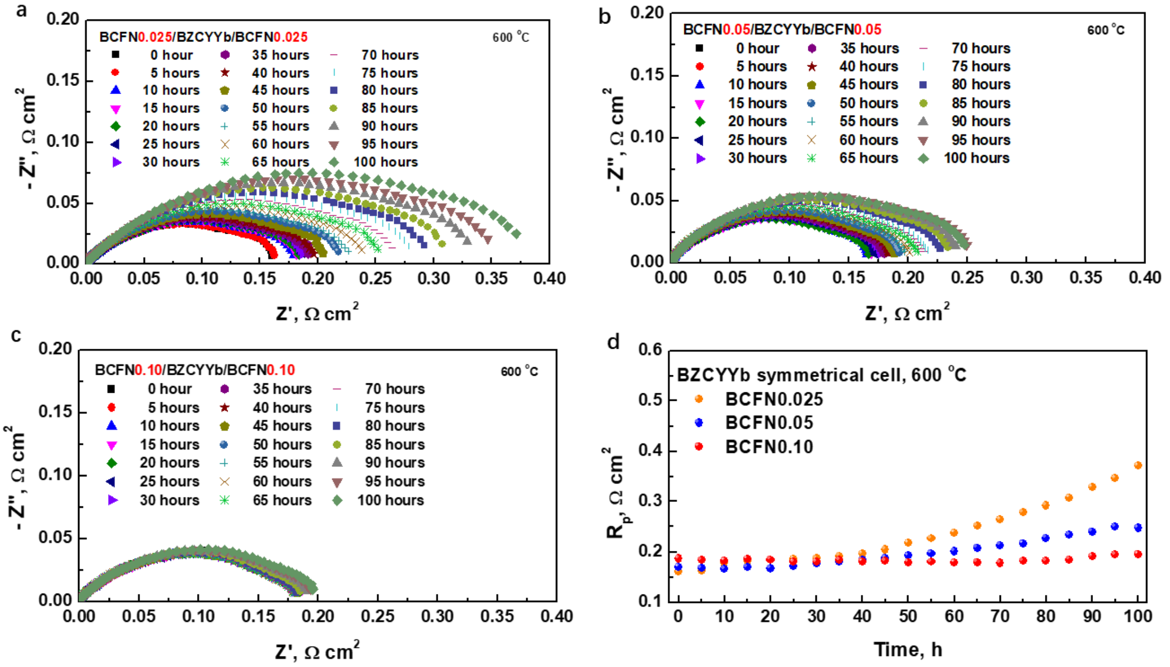

Supplementary Figure 9 Effect of Nb content in $Ba_{0.9}Co_{0.7}Fe_{0.2}Nb_xO_3$ (x=0.025, 0.05, and 0.1, denoted as BCFNx) on the electrode activity and durability. Electrochemical impedance spectra of BCFNx at different times and 600 °C: x=0.025 (a); x=0.05 (b); and x=0.1 (c); (d) R_p changes as a function of testing time in a period of 100h.

Supplementary Discussion: The electrode system (Nb-rich BCFN electrode covered by Nb-deficient BCFN NPs) showed a higher reaction activity compared to the single-phase Nb-deficient BCFN, as shown in the manuscript. In order to understand the effect of Nb content in the BCFN system on the activity and stability of $Ba_{0.9}Co_{0.7}Fe_{0.2}Nb_x$ (x=0.025, 0.05, and 0.1, denoted as BCFNx), we prepared BZCYYb electrolyte based symmetrical cells with BCFNx electrode. Shown in **Supplementary Figure 9** are the impedance spectra of BCFNx and the evolution of Rp value as a function of testing time. It is shown that BCFNx with less Nb showed a higher activity but worse durability, while BCFN with more Nb showed a lower activity but better durability. It has been reported that the *in-situ* formation of the Nb-deficient BCFN nanoparticles on BCFN electrodes is expected to have better structural stability since the risk of agglomeration is greatly reduced, thus enhancing the durability and thermal stability of the electrode. Such enhanced stability is also observed in other reports[1-4]. In addition, the nano-particles maintained a crystallographic coherence with the host perovskite lattice and consequently may experience lattice strain[2,5,6]. Such confined and seemingly anchored particles

are expected to exhibit considerably different physical and chemical properties as compared with the unconstrained particles, introduced by the traditional solution infiltration process[7,8].

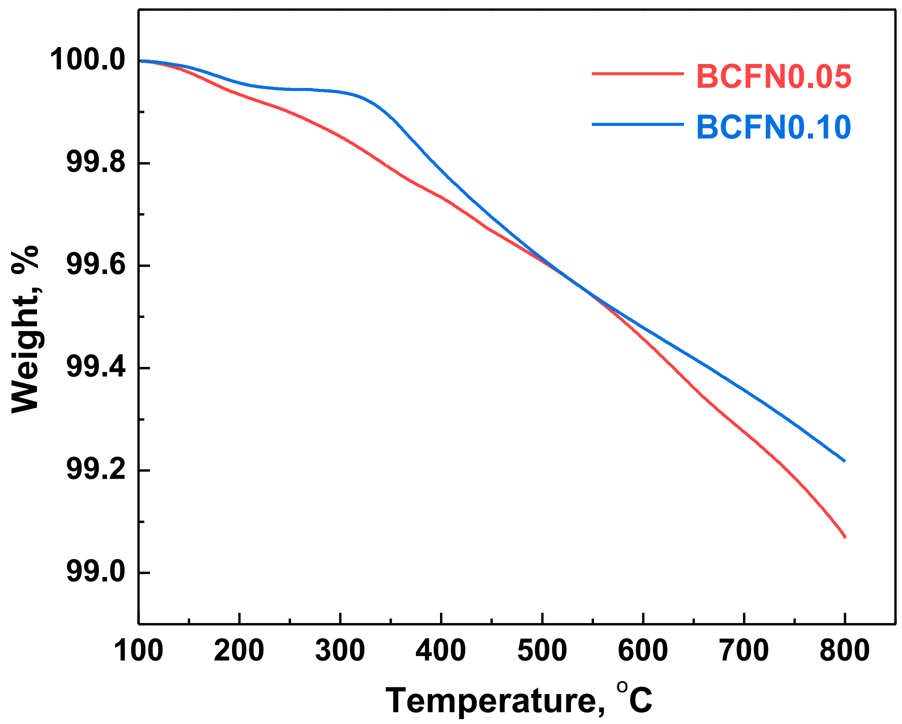

Supplementary Figure 10 Thermalgravimetric analyses of BCFN0.05 and BCFN0.1.

Supplementary Discussion: Nb-deficient BCFN (for example, $Ba_{0.9}Co_{0.7}Fe_{0.2}Nb_{0.05}O_3$) shows higher activity than BCFN with no deficiency ($Ba_{0.9}Co_{0.7}Fe_{0.2}Nb_{0.1}O_3$). It is reported that less reducible cations such as Zr, Ta, or Nb are expected to enhance the durability of perovskite materials[9,10] An Nb-deficient BCFN material on the surface is expected to have a higher concentration of oxygen vacancy than BCFN in the bulk (without an Nb deficiency), as confirmed by thermogravimetric analyses of BCFN0.1 and BCFN0.05 (**Supplementary Figure 10**). The valence changes of Co or Fe would provide more oxygen vacancies (more weight loss) at high temperatures in the oxygen reduction or evolution reactions, which is likely the reason for the higher activity of Nb-deficient BCFN nanoparticles.

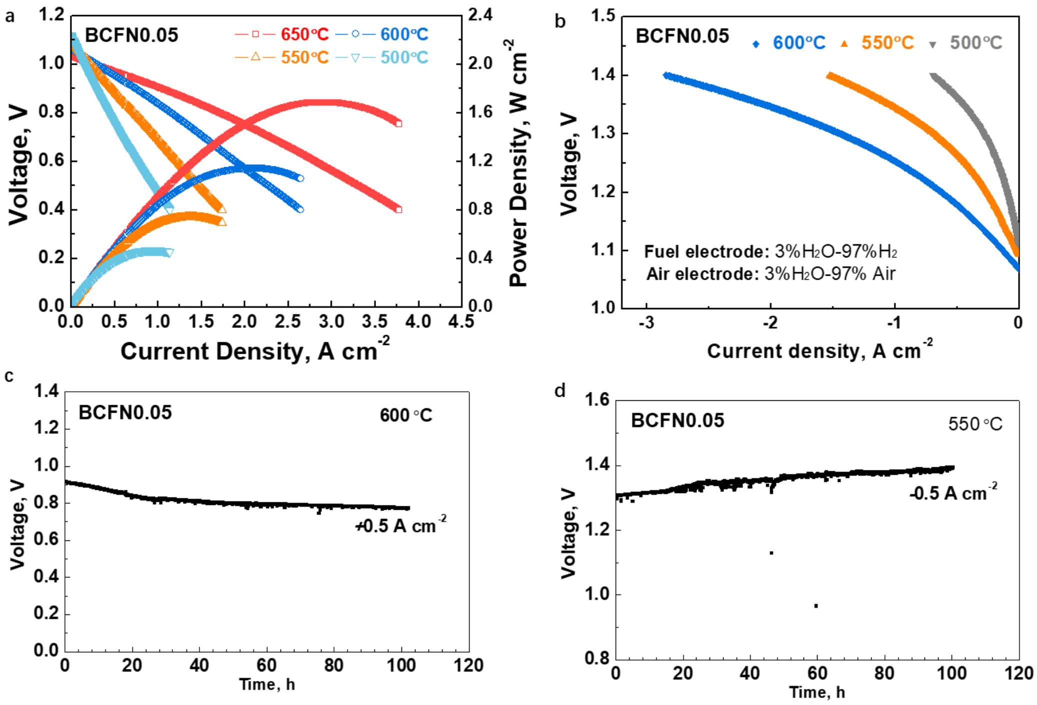

Supplementary Figure 11 Performance of a full cell with a BCFN0.05 air electrode. **(a)** Typical IVP curves in a fuel cell mode measured at 500-650 °C; **(b)** Typical IV curve in an electrolysis mode measured at 500-600 °C; **(c)** A short-term durability test of a fuel cell tested at 600 °C and a current density of +0.5 A cm^{-2}. and **(d)** A short-term durability test of an electrolysis cell tested at 550 °C and a current density of -0.5 A cm^{-2}

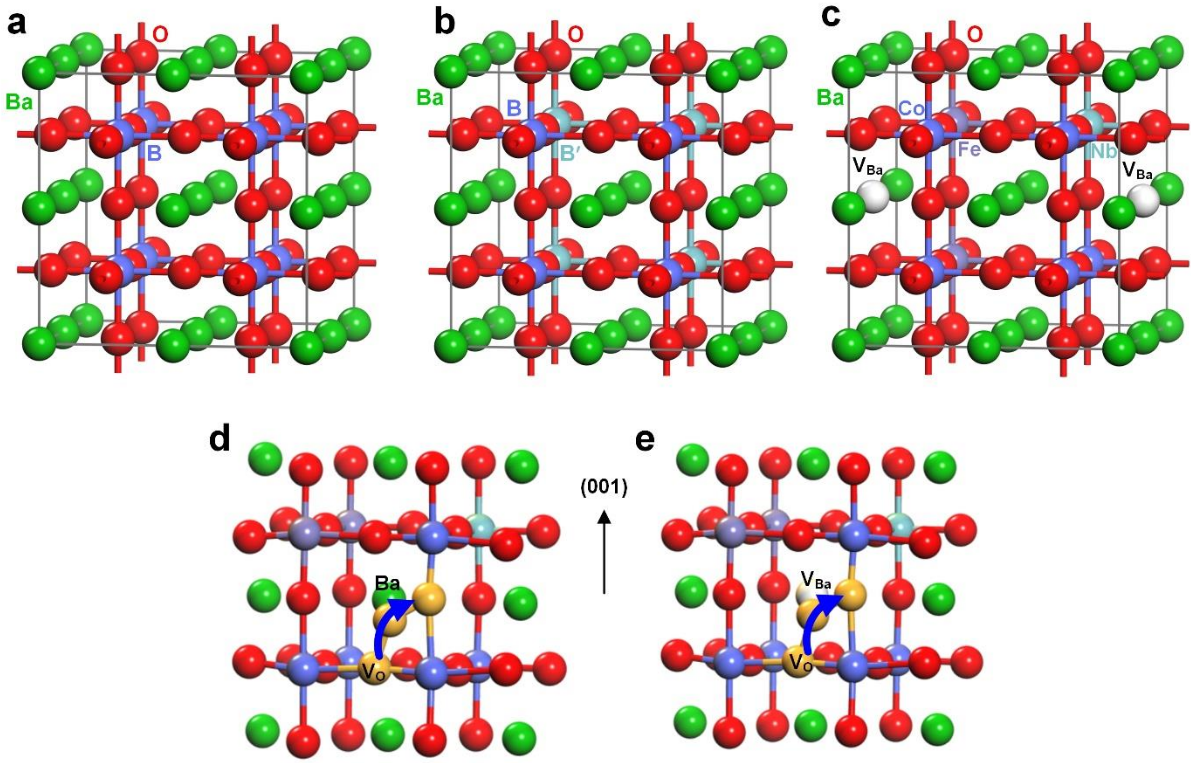

Supplementary Figure 12. Optimized bulk structures of **(a)** stoichiometric $BaBO_3$ perovskites with a cubic structure ($Pm\overline{3}m$) (8 Ba, 8 B, 24 O atoms) (B = Co, Fe, or Nb), **(b)** stoichiometric $Ba(B_{0.5}B'_{0.5})O_3$ perovskites with a cubic structure ($Pm\overline{3}m$) (B, B′ = Co, Fe, or Nb; 8 Ba, 4 B, 4 B′, and 24 O atoms), and **(c)** A-cation deficient $Ba_{0.9}(Co_{0.63}Fe_{0.25}Nb_{0.13})O_{3.0}$ with a cubic structure ($Pm\overline{3}m$) (7 Ba, 5 Co, 2 Fe, 1 Nb, 24 O atoms). V_{Ba} is a Ba vacancy. Schematic illustration of bulk diffusion of oxygen vacancies along the [001] direction of **(d)** $Ba(Co_{0.63}Fe_{0.25}Nb_{0.13})O_{3.0}$ with an migration energy of 0.58 eV and **(e)** A-cation deficient $Ba_{0.9}(Co_{0.63}Fe_{0.25}Nb_{0.13})O_{3.0}$ with that of 0.60 eV. V_O is an oxygen vacancy.

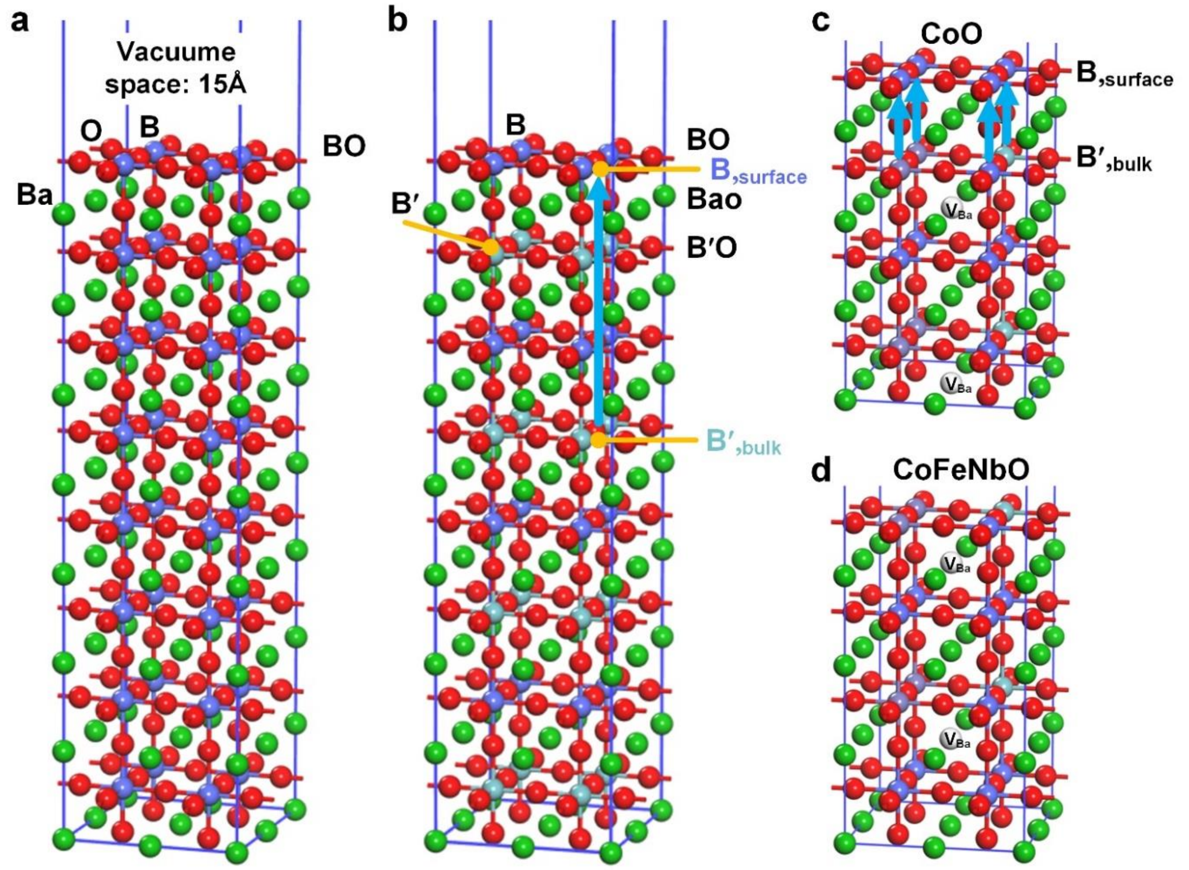

Supplementary Figure 13. 2-D slab models of (a) $BaBO_3$(001) (B = Co, Fe, or Nb) (32 Ba, 32 B, O 96 atoms) and (b) $Ba(B_{0.5}B'_{0.5})O_3$(001) (B, B′ = Co, Fe, or Nb) (32 Ba, 16 B, 16 B′, and 96 O atoms). (c) CoO-terminated and (d) CoFeNbO-terminated $Ba_{0.9}(Co_{0.63}Fe_{0.25}Nb_{0.13})O_{3.0}$(001) (14 Ba, 10 Co, 4 Fe, 2 Nb, 48 O atoms). V_{Ba} is a Ba vacancy. Note that the CoO-terminated BCFN(001) surface was used to examine the segregation energy from the bulk ($B'_{,bulk}$) to the surface ($B_{,surface}$) (*i.e.*, Co, Fe, or Nb to Co).

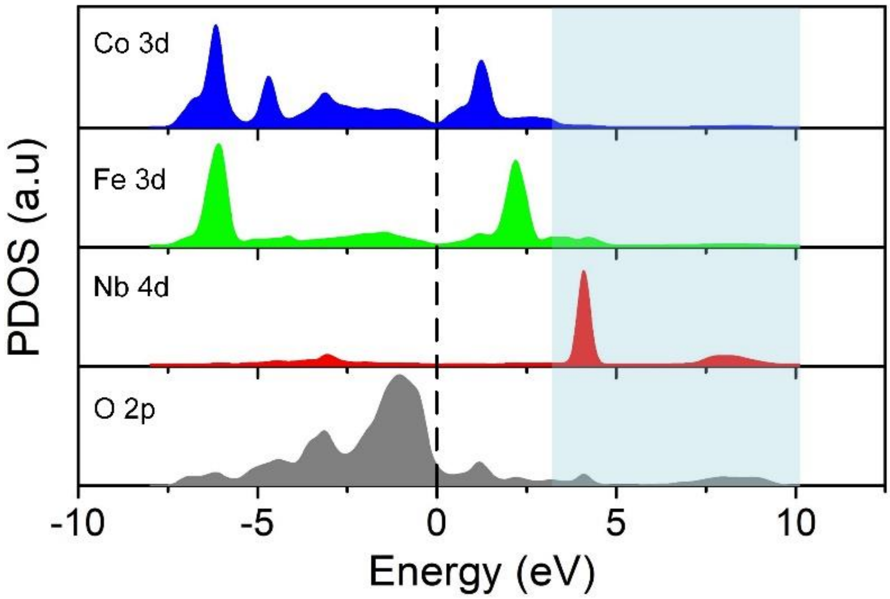

Supplementary Figure 14. Projected density of states (PDOSs) of the d-orbitals of Co, Fe, and Nb and the p-orbitals of O for $Ba_{0.9}(Co_{0.63}Fe_{0.25}Nb_{0.13})O_{3.0}$. The Fermi energy level (E_F) is set to zero.

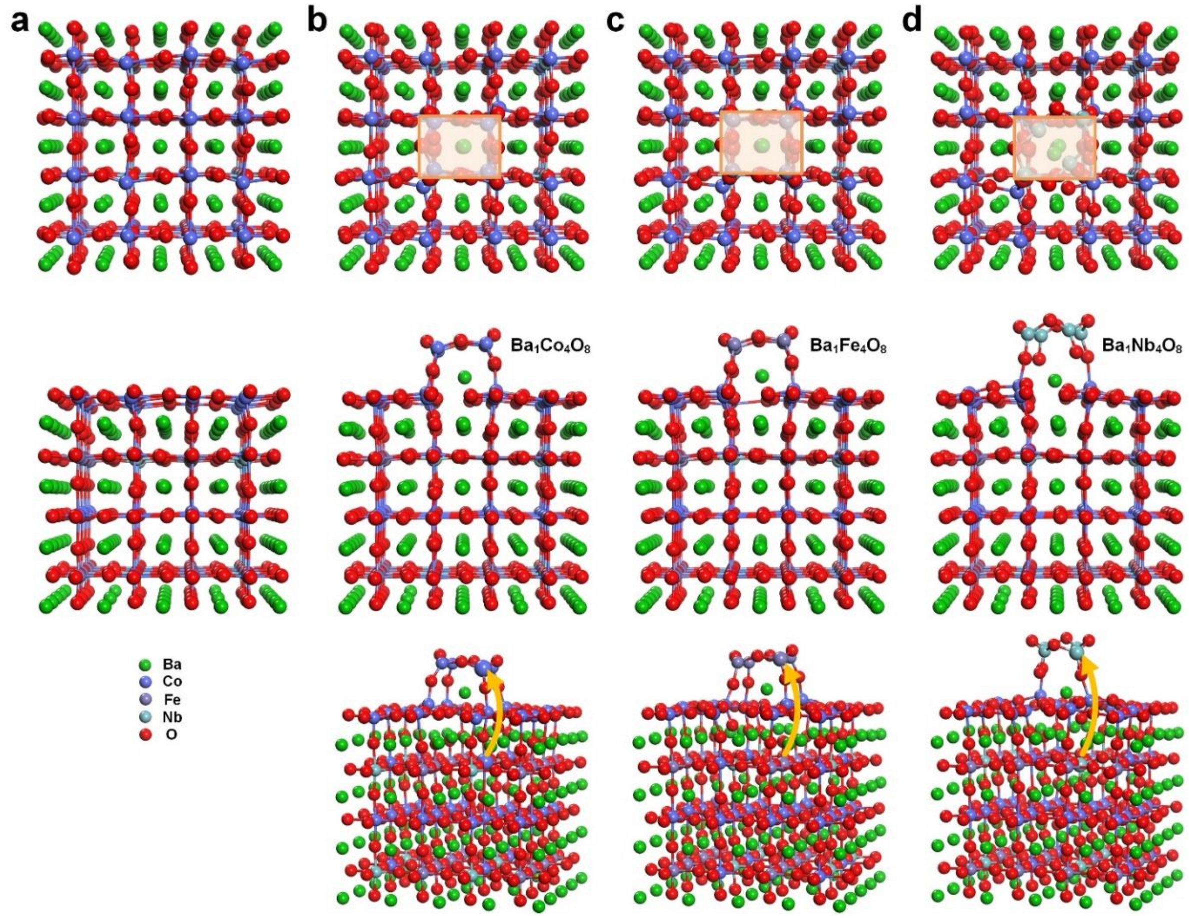

Supplementary Figure 15. 2-D slab models of (a) surface, (b) $Ba_1Co_4O_8$ NP, (c) $Ba_1Fe_4O_8$ NP, and (d) $Ba_1Nb_4O_8$ NP on BCFN(001) (56 Ba, 40 Co, 16 Fe, 8 Nb, 192 O atoms). The rectangular boxes are $Ba_1B_4O_8$ nanoparticles (B = Co, Fe, or Nb), while the arrows represent the segregation of a B cation from the bulk to the nanoparticle surface.

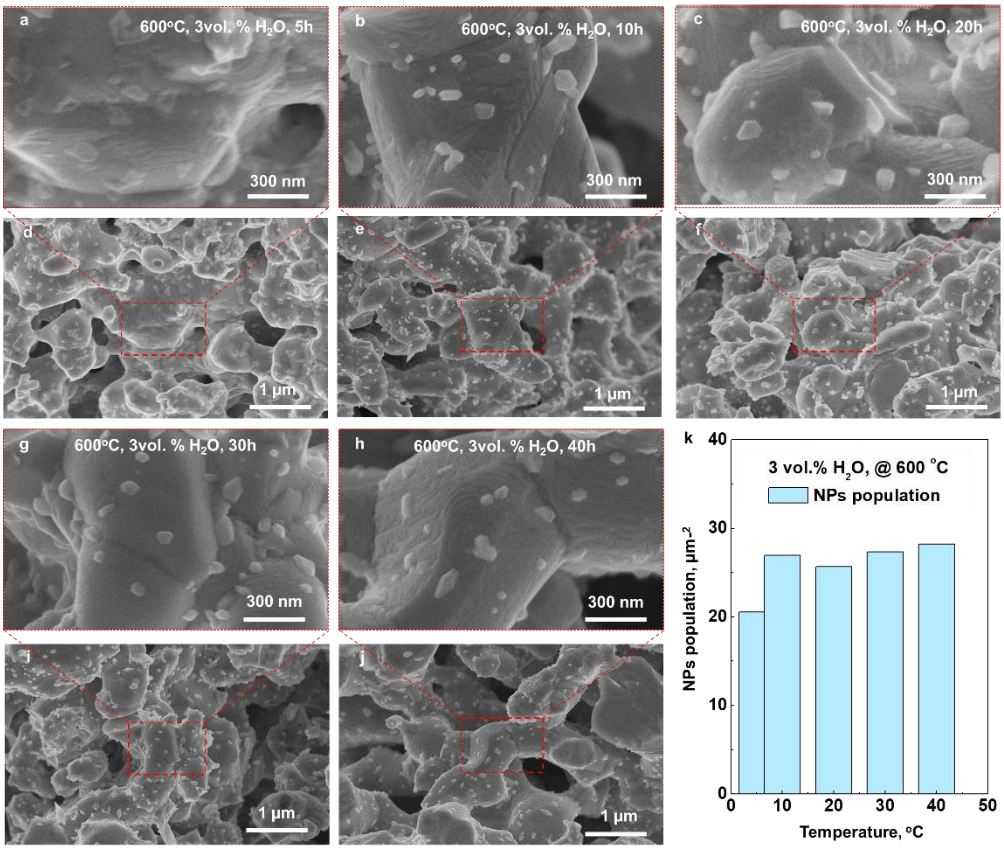

Supplementary Figure 16. Detailed SEM images of BCFN air electrode after operated under 3% humidified air at 600 ºC for 5 hours (a) & (d), 10 hours (b) & (e), 20 hours (c) & (f), 30 hours (g) & (i), 40 hours (h) & (j), respectively. (k) Time dependence of exsolved nanoparticles populations of BCFN air electrode operated at 3% humidified H_2O at 600 ºC.

Supplementary Discussion: First, we operated the BCFN air electrode at 600 ºC in the air with 3% H_2O for different durations (5-40 hours) at OCV conditions to disclose the relationship between the amount of exsolved nanoparticles and operating time (**Supplementary Figure 16**). As shown, a considerable number of nanoparticles (with a population of ~20 μm^{-2}) have been exsolved after 5 hours of treatment. The precipitated nanoparticles significantly increase as a function of treating time (in the following 35h). However, after 10h, the NP population tends to be stable.

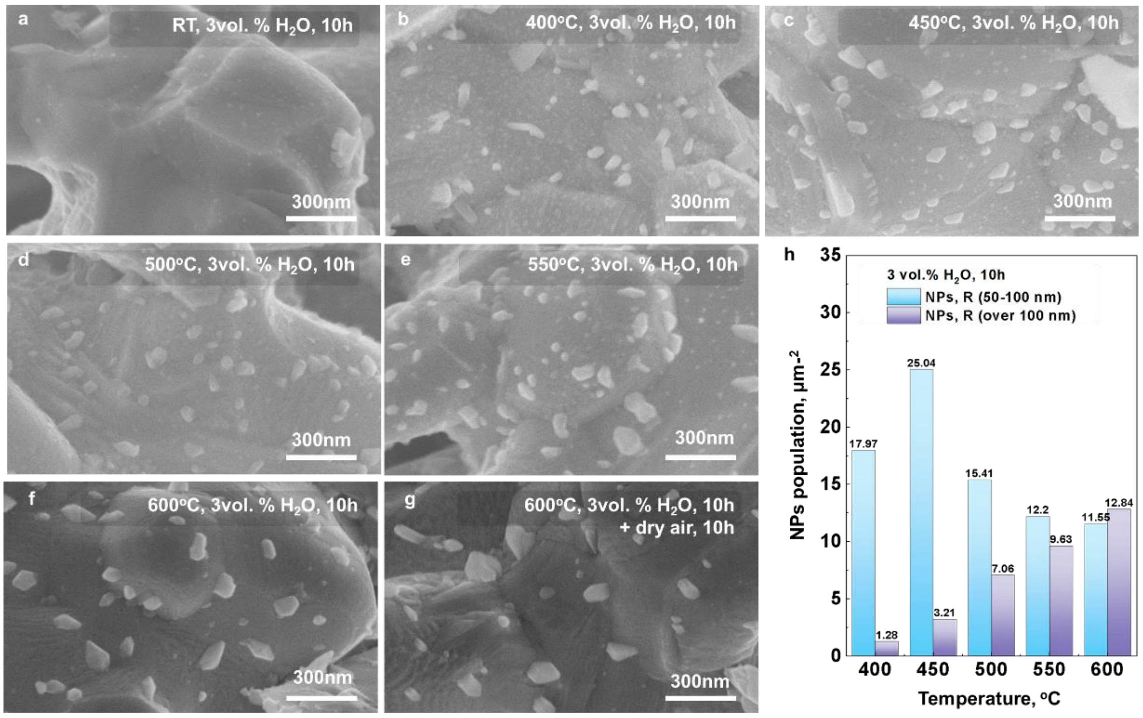

Supplementary Figure 17 Detailed SEM images of BCFN air electrode after operated under 3% humidified air for 10 hours at (**a**) room temperature, (**b**) 400 °C, (**c**) 450 °C, (**d**) 500 °C , (**e**) 550 °C and (**f**) 600 °C, respectively. (**g**) Typical SEM images of BCFN air electrode after operated with 3% H_2O humidified air at 600 °C for 10 hours and followed operated with dry air at 600 °C for 10 hours. Temperature-dependent exsolved nanoparticles population of BCFN air electrode operated at 3% H_2O humidified air for 10 hours. (**h**) populations of the exsolved nanoparticles with a diameter of 50-100 nm (blue) and over 100 nm (purple) when BCFN electrode was operated at 3% humidified H_2O for 10 hours at 400-600 °C.

Supplementary Discussion: In order to investigate the specific temperature that nanoparticles start to exsolve from the skeleton, we operated the BCFN air electrode at different temperatures (400-600 °C) with 3% humidified air for 10 hours at OCV condition (**Supplementary Figure 17**). It is revealed that BCFN approximately started to exsolve at 400 °C. Similarly, we roughly estimated the population of the exsolved nanoparticles (NP) and found that the NP population (over 100nm) increased significantly as temperature increased.

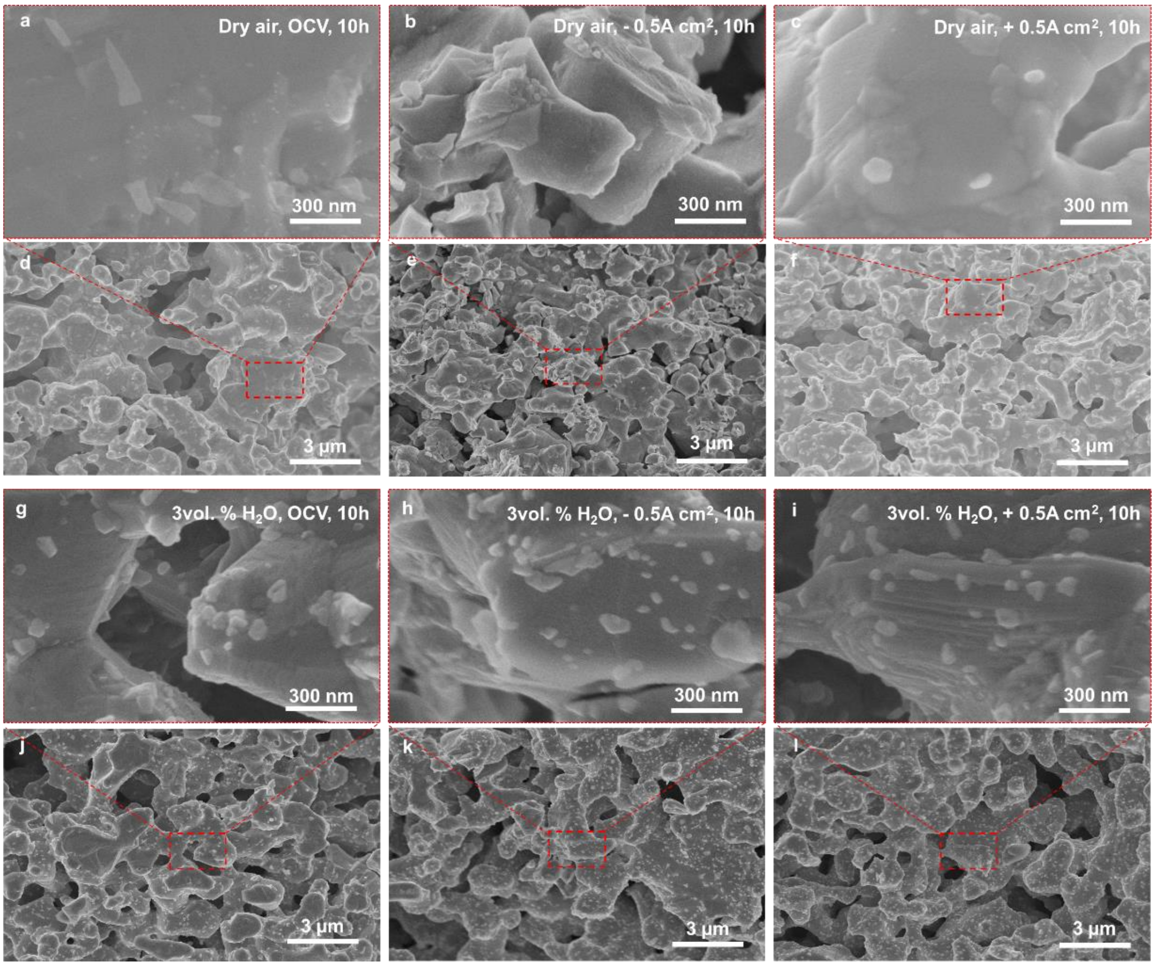

Supplementary Figure 18. Typical SEM images of BCFN air electrode after operated at OCV condition with wet (3% H_2O) hydrogen as fuel and dry air as oxidant **(a)** & **(d),** a current density of -0.5 A cm^{-2} with wet (3% H_2O) hydrogen as fuel and dry air as oxidant **(b)** & **(e),** and a current density of +0.5 A cm^{-2} with wet (3% H_2O) hydrogen as fuel and dry air as oxidant **(c)** & **(f)** at 600 °C for 10 hours; Typical SEM images of BCFN air electrode after operated at OCV condition with wet (3% H_2O) hydrogen as fuel and wet (3% H_2O) air as oxidant **(g)** & **(j),** a current density of -0.5 A cm^{-2} with wet (3% H_2O) hydrogen as fuel and wet (3% H_2O) air as oxidant **(h)** & **(k)** and a current density of +0.5 A cm^{-2} with wet (3% H_2O) hydrogen as fuel and wet (3% H_2O) air as oxidant **(i)** & **(l)** at 600 °C for 10 hours.

Supplementary Discussion: To evaluate if the voltage/current or the chemical potential of steam is the main reason for the exsolution, we treated the air electrode under the following conditions. First, we operated cells with BCFN air electrode at OCV condition when the air

electrode is exposed to dry air at 600 °C for 10 hours at current densities of 0, -0.5, and +0.5 Acm^{-2}. It is shown that very few nanoparticles are exsolved (**Supplementary Figure 18**a-f). In contrast, when the air electrode is exposed to wet air (3% H_2O) at current densities of 0, -0.5, and +0.5 Acm^{-2}, a great number of nanoparticles were observed on the skeleton (**Supplementary Figure 18g-l**), indicating that steam can lead to the exsolution. Therefore, it is reasonable to conclude that the current flowing may not be the factor required to induce the exsolution. The steam is most likely the main reason for the exsolution.

Supplementary Table 1. Temperature dependence of the polarization resistance (R_p) of BZCYYb symmetrical cell with BCFN and other high-performance electrodes reported recently.

Cathode	Temp. [°C]	R_p [Ω cm^{-2}]	Authors, Year
**$Ba_{0.9}Co_{0.7}Fe_{0.2}Nb_{0.1}O_{3-\delta}$ (BCFN)**	700	0.04153	**This work**
	650	0.10028	
	600	0.19689	
	550	0.47147	
	500	1.19746	
**$Nd_{0.5}Ba_{0.5}Fe_{0.9}Co_{0.1}O_{3-\delta}$ (NBFC)**	750	0.126	Lyagaeva, J., *et al, 2018*[11]
	700	0.178	
	650	0.390	
	600	1.000	
**$BaCo_{0.4}Fe_{0.4}Zr_{0.2}O_{3-\delta}$ (BCFZ)**	650	0.60	Shang, M., *et al, 2013*[12]
	600	1.00	
	550	1.80	
**$Ba_{0.5}Sr_{0.5}Co_{0.8}Fe_{0.2}O_{3-\delta}$ (BSCF)**	700	0.794	Bi, L., *et al, 2012*[13]
	650	1.318	
	600	1.990	
	550	3.981	
**$Ba_{0.5}Sr_{0.5}Co_{0.72}Fe_{0.18}Ti_{0.10}O_{3-\delta}$ (BSCFT)**	700	0.891	Meng, Y., *et al., 2021*[14]
	650	1.580	
	600	3.160	
	550	4.460	
**$BaCo_{0.4}Fe_{0.4}Zr_{0.1}Y_{0.1}O_{3-\delta}$ (BCFZY)**	700	0.10	Matsui, T., *et al., 2021*[15]
	650	0.40	
	600	0.85	
	550	1.15	
**$Ba_5SrSm_2Co_4O_{15}$ (BSSC)**	700	0.40	Huan, D., *et al., 2020*[16]
	650	0.68	
	600	1.20	

	550	2.20	
**$Sr_3EuFe_{2.5}Co_{0.5}O_{10-\delta}$ (SEFC)**	700<br>650<br>600	0.341<br>0.950<br>2.800	Huan, D., *et al., 2018*[17]
**$Ba(Co_{0.4}Fe_{0.4}Zr_{0.1}Y_{0.1})_{0.95}O_{3-\delta}$ ($BCFZY_{0.95}$)**	650<br>600<br>550	0.2<br>0.3<br>0.8	He, F., *et al., 2020*[18]
**$SrCo_{0.8}Fe_{0.15}Zr_{0.05}O_{3-\delta}$ (SCFZ)**	700<br>650<br>600<br>550<br>500	0.07<br>0.15<br>0.38<br>1.01<br>2.42	Lv, X., ., *et al., 2020*[19]
**$BaCo_{0.4}Fe_{0.4}Zr_{0.1}Y_{0.1}O_{3-\delta}$ (BCFZY)**	500	1.75	Liang, M., *et al., 2021*[20]
**$Ba(Co_{0.4}Fe_{0.4}Zr_{0.1}Y_{0.1})_{0.95}Ni_{0.05}O_{3-\delta}$ (BCFZYN)**	500	1.5	Liang, M., *et al., 2021*[20]
**$Ba(Co_{0.4}Fe_{0.4}Zr_{0.1}Y_{0.1})_{0.95}Zn_{0.05}O_{3-\delta}$ (BCFZYZ)**	500	1.83	Liang, M., *et al., 2021*[20]
**$Ba(Co_{0.4}Fe_{0.4}Zr_{0.1}Y_{0.1})_{0.95}Mn_{0.05}O_{3-\delta}$ (BCFZYM)**	500	2.38	Liang, M., *et al., 2021*[20]
**$Ba(Co_{0.4}Fe_{0.4}Zr_{0.1}Y_{0.1})_{0.95}Cu_{0.05}O_{3-\delta}$ (BCFZYC)**	500	1.70	Liang, M., *et al., 2021*[20]
**$Ba_{0.95}La_{0.05}Fe_{0.8}Zn_{0.2}O_{3-\delta}$ - $BaZr_{0.1}Ce_{0.7}Y_{0.1}Yb_{0.1}O_{3-\delta}$ (BLFZ–BZCYYb composite)**	750<br>700<br>650	0.159<br>0.297<br>0.683	Wang, Z., *et al., 2020*[21]
**$Ba_{0.95}Ca_{0.05}Co_{0.4}Fe_{0.4}Zr_{0.1}Y_{0.1}O_{3-\delta}$ (BCCFZY)**	750<br>700<br>650	0.290<br>0.631<br>1.488	Li, J., *et al., 2020*[22]
**$PrBa_{0.9}Co_{1.96}Nb_{0.04}O_{5+\delta}$ (PBCN)**	750<br>700<br>650<br>600	0.056<br>0.090<br>0.220<br>0.550	Zhang, W., *et al., 2021*[23]

Supplementary Table 2. Performance comparison of our cells and other high-performance cells reported recently.

Cathode	Electrolyte	Anode	Electrolyte thickness [μm]	Temp. [^oC]	P_{max} [W cm^{-2}]	Authors, Year
**$Ba_{0.5}Sr_{0.5}Co_{0.8}Fe_{0.2}O_{3-\delta}$**	$BaCe_{0.55}Zr_{0.3}Y_{0.15}O_{3-\delta}$ ($BCZY_3$)	NiO-$BCZY_3$	5	650<br>600<br>550	1.610<br>1.302<br>0.920	An et al.,2018[24]
**$PrBa_{0.5}Sr_{0.5}Co_{1.5}Fe_{0.5}O_{5+\delta}$ (PBSCF)**	$BaZr_{0.4}Ce_{0.4}Y_{0.1}Yb_{0.1}O_3$ (BZCYYb4411)	NiO-BZCYYb4411	15	600	0.8	Choi et al.,2018[25]
**$PrBa_{0.5}Sr_{0.5}Co_{1.5}Fe_{0.5}O_{5+\delta}$ (PBSCF) PLD layer**	$BaZr_{0.4}Ce_{0.4}Y_{0.1}Yb_{0.1}O_3$ (BZCYYb4411)	NiO-$BaZr_{0.4}Ce_{0.4}Y_{0.1}Yb_{0.1}O_3$ (BZCYYb4411)	15	650<br>600	1.4<br>1.1	Choi et al.,2018[25]
**BCZY631-$BaCo_{0.4}Fe_{0.4}Zr_{0.1}Y_{0.1}O_{3-\delta}$ ($BCFZY_{0.1}$)BCFZY**	BCZYYb4411+1wt % NiO	NiO-BCZYYb4411	15 - 50	600<br>550	0.648<br>0.515	Duan et al.,2015[26]
**$NdBa_{0.75}Ca_{0.25}Co_2O_{5+\delta}$ (NBCaC)-BZCYYb1711**	$BaZr_{0.1}Ce_{0.7}Y_{0.1}Yb_{0.1}O_3$ (BZCYYb1711)	Ni_4Co-$PrBaMn_2O_{5+\delta}$(PBM)-NiO-BZCYYb1711	15	700	1.48	Hua et al.,2016[27]
**$NdBa_{0.5}Sr_{0.5}Co_{1.5}Fe_{0.5}O_{5+\delta}$(NBSCF)**	BCZYYb1711	NiO-BCZYYb1711	14.7	700<br>600	2.1<br>0.69	Kim et al.,2014[28]
**$PrBa_{0.5}Sr_{0.5}Co_{1.5}Fe_{0.5}O_{5+\delta}$ (PBSCF)**	BCZYYb1711	NiO-BCZYYb1711	14.1	700<br>650<br>600	1.374<br>1.048<br>0.704	Seong et al.,2018[29]
**$La_{0.6}Sr_{0.4}CoO_{3-\delta}$(LSC)**	$BaCe_{0.55}Zr_{0.3}Y_{0.15}O_{3-\delta}$ ($BCZY_3$)	NiO-BCZY, NiO-YSZ	1	650<br>600<br>550	1.3<br>1.1<br>0.8	Bae et al.,2018[30]

**$La_{0.6}Sr_{0.4}Co_{0.2}Fe_{0.8}O_{3-\delta}$(LSCF)**	BCZYYb1711	NiO-BCZYYb1711	10	600	1.125	Yang et al.,2009[31]
**$BaCo_{0.4}Fe_{0.4}Zr_{0.2}O_{3-\delta}$**	BCZYYb1711	NiO-BCZYYb1711	30	750<br>650	1.1<br>0.66	Shang et al.,2013[32]
**BCZY-LSCF**	BCZYYb1711	NiO-BCZYYb1711	14.7	600	0.225	Kim et al.,2014[28]
**$Ba_{0.5}Sr_{0.5}Co_{0.8}Fe_{0.2}O_{3-\delta}$**	$BaCe_{0.55}Zr_{0.3}Y_{0.15}O_{3-\delta}$ ($BCZY_3$)	NiO-$BCZY_3$	1.5	600	0.508	Bae et al.,2016[33]
**$La_{0.6}Sr_{0.4}CoO_{3-\delta}$(LSC)**	$BaCe_{0.55}Zr_{0.3}Y_{0.15}O_{3-\delta}$ ($BCZY_3$)	NiO-BCZY	5	600	0.74	Bae et al.,2017[34]
**BCZY**	BCZYYb1711	NiO-BCZYYb1711	7	550	0.44	Braun et al.,2019[35]
**$BaCo_{0.7}(Ce_{0.8}Y_{0.2})_{0.3}O_{3-\delta}$(BCCY)**	BCZYYb1711	NiO-BCZYYb1711	16.1	650<br>600	0.993<br>0.73	Song et al.,2019[36]
**$PrCo_{0.5}Ni_{0.5}O_{3-\delta}$ nano-fiber**	BCZYYb4411	NiO-BCZYYb4411	10	600<br>550	0.607<br>0.444	Ding et al.,2020[37]
**$PrNi_{0.5}Mn_{0.5}O_{3-\delta}$ (PNM)**	BCZYYb1711	NiO-BCZYYb1711	30	700<br>650	0.8<br>0.441	Chen et al.,2018[38]
**$PrBa_{0.5}Sr_{0.5}Co_{1.5}Fe_{0.5}O_{5+\delta}$ (PBSCF)**	BCZYYb4411	NiO-BCZYYb4411	10	650<br>600<br>550	1.9<br>1.45<br>1.01	Choi et al.,2021[39]
**$PrBa_{0.5}Sr_{0.5}Co_{1.5}Fe_{0.5}O_{5+\delta}$ (PBSCF)**	BCZYYb1711	NiO-BCZYYb1711	10	700<br>650<br>600<br>550	1.609<br>1.257<br>0.723<br>0.360	Hua et al.,2021[40]
**$Ba_{0.9}Co_{0.7}Fe_{0.2}Nb_{0.1}O_{3-\delta}$**	BCZYYb1711	NiO-BCZYYb1711	10	650<br>600<br>550<br>500	1.707<br>1.207<br>0.820<br>0.550	**This work**

Supplementary Table 3. Performance comparison of our cell and other cells reported by others.

Cathode	Electrolyte	Anode	Electrolyte thickness [μm]	Temp. [^oC]	Current Density@1.3 V [A cm^{-2}]	Authors, Year
**$PrBa_{0.5}Sr_{0.5}Co_{1.5}Fe_{0.5}O_{5+\delta}$(PBSCF)**	$BaZr_{0.4}Ce_{0.4}Y_{0.1}Yb_{0.1}O_3$ (BZCYYb4411)	NiO-BZCYYb4411	15	650<br>600	-2.208<br>-1.926	Choi et al.,2019[41]
**$NdBa_{0.5}Sr_{0.5}Co_{1.5}Fe_{0.5}O_{5+\delta}$(NBSCF)-BZCYYb**	BCZYYb1711	NiO-BCZYYb1711	14.7	650<br>600<br>550	-1.705<br>-0.805<br>-0.399	Kim et al.,2018[42]
**$PrBa_{0.5}Sr_{0.5}Co_{1.5}Fe_{0.5}O_{5+\delta}$(PBSCF)**	BaHfCeYYbO (BHCYYb3511)	Ni-BHCYYb (3511)	15	650<br>600	-0.695<br>-0.285	Murphy et al.,2020[43]
**Pr_2NiO_4-BZCY**	$BaZr_{0.2}Ce_{0.6}Y_{0.2}O_3$ (BZCY)	Ni-BZCY	20	650<br>600<br>550	-0.604<br>-0.349<br>-0.221	Li et al.,2018[44]
**$PrCo_{0.5}Ni_{0.5}O_{3-\delta}$ nano-fiber**	BCZYYb4411	NiO-BCZYYb4411	$PrCo_{0.5}Ni_{0.5}O_{3-\delta}$ nano-fiber	600<br>550	-1.000<br>-0.698	Ding et al.,2020[37]
**$PrBa_{0.5}Sr_{0.5}Co_{1.5}Fe_{0.5}O_{5+\delta}$(PBSCF)**	$BaZr_{0.8}Y_{0.2}Yb_{0.1}O_3$ (BZY20)	NiO-BZY20	15	600<br>550<br>500	-0.903<br>-0.708<br>-0.500	Duan et al.,2019[45]
**$BaGd_{0.8}La_{0.2}Co_2O_{6-\delta}$**	$BaZr_{0.2}Ce_{0.7}Y_{0.1}O_3$ (BZCY)	Ni-BZCY	25	600<br>500	-0.131<br>-0.074	Vøllestad et al.,2019[46]
**$Ba_{0.9}Co_{0.7}Fe_{0.2}Nb_{0.1}O_{3-\delta}$**	BCZYYb1711	NiO-BCZYYb1711	10	650<br>600<br>550<br>500	-2.735<br>-1.511<br>-0.718<br>-0.265	**This work**

Supplementary Table 4. Optimized lattice parameters (a_0) of $BaBO_3$ (B = Co, Fe, or Nb) and the surface energies of BaO- and BO-terminated $BaBO_3$(001).

	bulk	slab	
	a_0 (Å)	surface energy (J/m^2)	termination
$BaCoO_3$	3.9562	0.45	BaO
		0.51	CoO
$BaFeO_3$	3.9761	0.81	BaO
		0.82	FeO
$BaNbO_3$	4.1208	0.94	BaO
		0.93	NbO
Surface stability of BO termination			CoO > FeO > NbO

Note: In this comparison, all layers were fully relaxed without any restriction. As shown in the summary of BaO-terminated surfaces, the stability may be dependent on B cation types.

Supplementary Table 5. Optimized lattice constants (a_0) of $Ba(B_{0.5}B'_{0.5})O_3$ (B, B′ = Co, Fe, or Nb) and the segregation energy of $Ba(B_{0.5}B'_{0.5})O_3(001)$.

	bulk	slab			
	a_0 (Å)	segregation atom	segregation energy (J/m^2)	termination	segregation tendency
$Ba(Co_{0.5}Fe_{0.5})O_3$	3.9725	Co	–0.14	CoO	Co > Fe
		Fe	+0.33	FeO	
$Ba(Co_{0.5}Nb_{0.5})O_3$	4.0581	Co	–0.54	CoO	Co > Nb
		Nb	+0.38	NbO	
$Ba(Fe_{0.5}Nb_{0.5})O_3$	4.0693	Fe	–0.57	FeO	Fe > Nb
		Nb	+1.13	NbO	
**Total segregation order**					Co > Fe > Nb

Supplementary Table 6. Optimized lattice constant (a_0) and oxygen vacancy formation energies (E_{OV}) of $Ba_{0.9}(Co_{0.63}Fe_{0.25}Nb_{0.13})O_{3.0}$ (BCFN) and surface energies of $Ba_{0.9}(Co_{0.63}Fe_{0.25}Nb_{0.13})O_{3.0}$(001) (BCFN(001)).

	bulk		slab	
	a_0 (Å)	E_{OV} (eV)[1]	termination	surface energy (J/m^2)[2]
$Ba_{0.9}(Co_{0.63}Fe_{0.25}Nb_{0.13})O_{3.0}$ (7 Ba, 5 Co, 2 Fe, 1 Nb, 24 O)	3.9768	2.95	CoO	0.78
			CoFeNbO	1.03
			BaO	0.93

Note 1: The oxygen vacancy formation energy was averaged after calculating at 24 oxygen vacancy positions. An oxygen vacancy formation energy of $Ba(Co_{0.63}Fe_{0.25}Nb_{0.13})O_{3.0}$ with a cubic structure (8 Ba, 5 Co, 2 Fe, 1 Nb, 24 O atoms) calculated is 1.87 eV, indicating ~37% more difficult to generate oxygen vacancies.

Note 2: It is an averaged surface energy of two BaO-terminated models (1. BaO-CoFeNbO-BaO-CoO- (0.91 J/m^2) and 2. BaO-CoO-BaO-CoFeNbO- (0.95 J/m^2)). The surface models were prepared with 16 layers, and the bottom eight layers were fixed at the bulk parameters.

Supplementary Table 7. Segregation energies of $Ba_{0.9}(Co_{0.63}Fe_{0.25}Nb_{0.13})O_{3.0}(001)$.

surface configuration[1]	segregation energy (eV)[2]				remark
	Co	Fe_1	Fe_2	Nb	
CoO	0.39	0.71	2.33	1.18	before swapping Co (surface) and Nb (bulk)
CoNbO	-	0.09	–0.04	-	before swapping Co (surface) and Nb (bulk)

1. The energy difference of CoNbO (after swapping Nb and Co atoms) and CoO (before switching Nb and Co atoms) is 0.17 J/m^2, indicating that Nb makes the surface less stable than the CoO-terminated surface.
2. The segregation energy of Fe becomes smaller after an Nb atom segregates to the surface. It explains that Nb that locally segregates to the surface augments the segregation of Fe.

Supplementary References

1. Kobsiriphat, W., *et al.* Nickel- and Ruthenium-Doped Lanthanum Chromite Anodes: Effects of Nanoscale Metal Precipitation on Solid Oxide Fuel Cell Performance. *J. Electrochem. Soc.* **157**, B279 (2010).
2. Neagu, D., *et al.* Nano-socketed nickel particles with enhanced coking resistance grown in situ by redox exsolution. *Nat. Commun.* **6**, 8120 (2015).
3. Kim, J.H., *et al.* Ex-Solved Ag Nanocatalysts on a Sr-Free Parent Scaffold Authorize a Highly Efficient Route of Oxygen Reduction. *Adv. Funct. Mater.* **30**, 2001326 (2020).
4. Kim, J.H., *et al.* Self-assembled nano-composite perovskites as highly efficient and robust hybrid cathodes for solid oxide fuel cells. *J. Mater. Chem. A* (2022).
5. Neagu, D., Tsekouras, G., Miller, D.N., Ménard, H. & Irvine, J.T.S. In situ growth of nanoparticles through control of non-stoichiometry. *Nat. Chem.* **5**, 916-923 (2013).
6. Kim, J.H., *et al.* Nanoparticle Ex-solution for Supported Catalysts: Materials Design, Mechanism and Future Perspectives. *ACS Nano* **15**, 81-110 (2021).
7. Irvine, J.T.S., *et al.* Evolution of the electrochemical interface in high-temperature fuel cells and electrolysers. *Nat. Energy* **1**, 15014 (2016).
8. Jiang, S.P. A review of wet impregnation--An alternative method for the fabrication of high performance and nano-structured electrodes of solid oxide fuel cells. *Mat. Sci. Eng.: A* **418**, 199-210 (2006).
9. Tsvetkov, N., Lu, Q., Sun, L., Crumlin, E.J. & Yildiz, B. Improved chemical and electrochemical stability of perovskite oxides with less reducible cations at the surface. *Nat. Mater.* **15**, 1010-1016 (2016).
10. Li, M., *et al.* A niobium and tantalum co-doped perovskite cathode for solid oxide fuel cells operating below 500 °C. *Nat. Commun.* **8**, 13990 (2017).
11. Lyagaeva, J., *et al.* Designing a protonic ceramic fuel cell with novel electrochemically active oxygen electrodes based on doped $Nd_{0.5}Ba_{0.5}FeO_{3-\delta}$. *Dalton T.* **47**, 8149-8157 (2018).
12. Shang, M., Tong, J. & O'Hayre, R. *RSC Adv.* 3, 15769 (2013).
13. Bi, L., Fabbri, E. & Traversa, E. Novel $Ba_{0.5}Sr_{0.5}(Co_{0.8}Fe_{0.2})_{1-x}Ti_xO_{3-\delta}$ (x= 0, 0.05 and 0.1) cathode materials for proton-conducting solid oxide fuel cells. *Solid State Ionics* **214**, 1-5 (2012).
14. Meng, Y., *et al.* Oxygen exchange and bulk diffusivity of $BaCo_{0.4}Fe_{0.4}Zr_{0.1}Y_{0.1}O_{3-\delta}$: Quantitative assessment of active cathode material for protonic ceramic fuel cells. *Solid State Ionics* **368**, 115639 (2021).
15. Matsui, T., *et al.* Oxygen reduction reaction over $(Ba, Sr)_6Re_2Co_4O_{15}$–Ba(Ce, Pr, Y) O_3 composite cathodes for proton-conducting ceramic fuel cells. *J. Mater. Chem. A* **9**, 15199-15206 (2021).
16. Huan, D., *et al.* A Durable Ruddlesden-Popper Cathode for Protonic Ceramic Fuel Cells. *ChemSusChem* **13**, 4994-5003 (2020).
17. Huan, D., *et al.* New, efficient, and reliable air electrode material for proton-conducting reversible solid oxide cells. *ACS Appl. Mater. Inter.* **10**, 1761-1770 (2018).
18. He, F., *et al.* High-performance proton-conducting fuel cell with b-site-deficient perovskites for all cell components. *Energy & Fuels* **34**, 11464-11471 (2020).
19. Lv, X., Chen, H., Zhou, W., Li, S.-D. & Shao, Z. A CO_2-tolerant $SrCo_{0.8}Fe_{0.15}Zr_{0.05}O_{3-\delta}$ cathode for proton-conducting solid oxide fuel cells. *J. Mater. Chem. A* **8**, 11292-11301 (2020).
20. Liang, M., *et al.* Nickel-doped $BaCo_{0.4}Fe_{0.4}Zr_{0.1}Y_{0.1}O_{3-\delta}$ as a new high-performance cathode for both oxygen-ion and proton conducting fuel cells. *Chem. Eng. J.* **420**, 127717 (2021).
21. Wang, Z., *et al.* $Ba_{0.95}La_{0.05}Fe_{0.8}Zn_{0.2}O_{3-\delta}$ cobalt-free perovskite as a triple-conducting cathode for proton-

conducting solid oxide fuel cells. *Ceram. Int.* **46**, 18216-18223 (2020).

22. Li, J., *et al.* Ca-containing $Ba_{0.95}Ca_{0.05}Co_{0.4}Fe_{0.4}Zr_{0.1}Y_{0.1}O_{3-\delta}$ cathode with high CO_2-poisoning tolerance for proton-conducting solid oxide fuel cells. *J. Power Sources* **453**, 227909 (2020).
23. Zhang, W., *et al.* A highly efficient and durable air electrode for intermediate-temperature reversible solid oxide cells. *Appl. Catal. B-Environ.* **299**, 120631 (2021).
24. An, H., *et al.* A 5×5 cm^2 protonic ceramic fuel cell with a power density of 1.3 W cm^{-2} at 600 ºC. *Nat. Energy* **3**, 870-875 (2018).
25. Choi, S., *et al.* Exceptional power density and stability at intermediate temperatures in protonic ceramic fuel cells. *Nat. Energy* **3**, 202-210 (2018).
26. Duan, C., *et al.* Readily processed protonic ceramic fuel cells with high performance at low temperatures. *Science* **349**, 1321-1326 (2015).
27. Hua, B., *et al.* Anode-Engineered Protonic Ceramic Fuel Cell with Excellent Performance and Fuel Compatibility. *Adv. mater.* **28**, 8922-8926 (2016).
28. Kim, J., *et al.* Triple-Conducting Layered Perovskites as Cathode Materials for Proton-Conducting Solid Oxide Fuel Cells. *ChemSusChem* **7**, 2811-2815 (2014).
29. Seong, A., *et al.* Influence of cathode porosity on high performance protonic ceramic fuel cells with $PrBa_{0.5}Sr_{0.5}Co_{1.5}Fe_{0.5}O_{5-\delta}$ cathode. *J. Electrochem. Soc.* **165**, F1098 (2018).
30. Bae, K., Kim, D.H., Choi, H.J., Son, J.W. & Shim, J.H. High-Performance Protonic Ceramic Fuel Cells with 1 μm Thick Y: Ba (Ce, Zr) O_3 Electrolytes. *Adv. Energy Mater.* **8**, 1801315 (2018).
31. Yang, L., *et al.* Enhanced sulfur and coking tolerance of a mixed ion conductor for SOFCs: $BaZr_{0.1}Ce_{0.7}Y_{0.2-x}Yb_xO_{3-\delta}$. *Science* **326**, 126-129 (2009).
32. Shang, M., Tong, J. & O'Hayre, R. *RSC Adv.* 3, 15769 (2013).
33. Bae, K., *et al.* High-performance thin-film protonic ceramic fuel cells fabricated on anode supports with a non-proton-conducting ceramic matrix. *J. Mater. Chem. A* **4**, 6395-6403 (2016).
34. Bae, K., *et al.* Demonstrating the potential of yttrium-doped barium zirconate electrolyte for high-performance fuel cells. *Nat. commun.* **8**, 1-9 (2017).
35. Braun, R.J., *et al.* Development of kW-scale protonic ceramic fuel cells and systems. *ECS Trans.* **91**, 997 (2019).
36. Song, Y., *et al.* Self-Assembled Triple-Conducting Nanocomposite as a Superior Protonic Ceramic Fuel Cell Cathode. *Joule* **3**, 2842-2853 (2019).
37. Ding, H., *et al.* Self-sustainable protonic ceramic electrochemical cells using a triple conducting electrode for hydrogen and power production. *Nat. commun.* **11**, 1-11 (2020).
38. Chen, Y., *et al.* An In Situ Formed, Dual-Phase Cathode with a Highly Active Catalyst Coating for Protonic Ceramic Fuel Cells. *Adv. Funct. Mater.* **28**, 1704907 (2018).
39. Choi, M., *et al.* Exceptionally high performance of protonic ceramic fuel cells with stoichiometric electrolytes. *Energ. Environ. Sci.* (2021).
40. Zhang, H., *et al.* An Efficient and Durable Anode for Ammonia Protonic Ceramic Fuel Cells. *Energy Environ. Sci.* , 15, 287-295 (2022).
41. Choi, S., Davenport, T.C. & Haile, S.M. Protonic ceramic electrochemical cells for hydrogen production and electricity generation: exceptional reversibility, stability, and demonstrated faradaic efficiency. *Energy Environ. Sci.* **12**, 206-215 (2019).
42. Kim, J., *et al.* Hybrid-solid oxide electrolysis cell: A new strategy for efficient hydrogen production. *Nano Energy* **44**, 121-126 (2018).
43. Murphy, R., *et al.* A New Family of Proton-Conducting Electrolytes for Reversible Solid Oxide Cells:

$BaHf_xCe_{0.8-x}Y_{0.1}Yb_{0.1}O_{3-\delta}$. *Adv. Funct. Mater.* **30**, 2002265 (2020).

44. Li, W., *et al.* High performing triple-conductive $Pr_2NiO_{4+\delta}$ anode for proton-conducting steam solid oxide electrolysis cell. *J. Mater. Chem. A* **6**, 18057-18066 (2018).

45. Duan, C., *et al.* Highly efficient reversible protonic ceramic electrochemical cells for power generation and fuel production. *Nat. Energy* **4**, 230-240 (2019).

46. Vøllestad, E., *et al.* Mixed proton and electron conducting double perovskite anodes for stable and efficient tubular proton ceramic electrolysers. *Nat. mater.* **18**, 752-759 (2019).
